# Association between serum cotinine and hepatic steatosis and liver fibrosis in adolescent: a population-based study in the United States

**DOI:** 10.1038/s41598-024-61771-3

**Published:** 2024-05-19

**Authors:** Dan She, Shangming Jiang, Siqi Yuan

**Affiliations:** Department of Clinical Laboratory, Union Hospital, Tongji Medical College, Huazhong University of Science and Technology, 430022 Wuhan, China

**Keywords:** Serum cotinine, NAFLD, NHANES, Hepatic steatosis, Liver fibrosis, Diseases, Endocrinology, Medical research, Risk factors

## Abstract

Tobacco exposure is known to be associated with a higher prevalence and incidence of liver diseases. Cotinine, a metabolite of nicotine, is a typical indicator of tobacco exposure. However, the relationship of serum cotinine levels with hepatic steatosis and liver fibrosis remains controversial and these relationships need more research to explored in American teenagers. Cross-sectional data included 1433 participants aged 12–19 from the National Health and Nutrition Examination Survey (NHANES) from 2017 to 2020 were thoroughly used for this study. The linear relationships between serum cotinine levels and the Liver Stiffness Measurement (LSM) and Controlled Attenuation Parameter (CAP) were examined using multiple linear regression models. Subgroup analysis, interaction tests, and nonlinear interactions were also carried out. Serum cotinine levels > 2.99 ng/ml [β = 0.41 (0.07, 0.76), *p* = 0.018] and 0.05–2.99 ng/ml [β = 0.24 (0.00, 0.49), *p* = 0.048] showed a significant positive connection with LSM in multivariate linear regression analysis when compared to serum cotinine levels ≤ 0.05 ng/ml (*p* for trend = 0.006). Moreover, we discovered an inverted U-shaped association of log2-transformed cotinine with LSM with an inflection point of 4.53 using a two-stage linear regression model. However, according to multiple regression analysis, serum cotinine and CAP did not significantly correlate (*p* = 0.512). In conclusion, this study demonstrated that smoking cessation and keep away from secondhand smoking may beneficial for liver health in American teenagers.

## Introduction

Non-alcoholic fatty liver disease (NAFLD) is a prevalent chronic liver disease globally, with a growing prevalence in teenagers^1,2^. NAFLD occurs around 10% among kids and up to 35% among obese youths^3,4^. The specific mechanisms causing NAFLD development in children are not entirely known, although risk factors include juvenile obesity, poor eating habits, and particular genetic susceptibility. Standard pathophysiologic processes in many liver illnesses include hepatic steatosis and liver fibrosis, which are frequently employed as markers to analyze the chronic liver disease and cirrhosis course and prognosis^5–7^. Observational investigations have found that both hepatic steatosis and fibrosis are related with an increased risk of all-cause mortality. Thus, assessing the degree of hepatic fibrosis and steatosis is essential for the clinical prognosis and assessment of NAFLD patients^8^. To assess hepatic steatosis and fibrosis, the Liver Stiffness Measurement (LSM) and Controlled Attenuation Parameter (CAP) were obtained from vibration-controlled transient elastography (VCTE)^9–11^.

Smoke exposure has shown a considerable correlation with adult and adolescent health problems^12,13^ such as cardiovascular diseases, cancer, and type 2 diabetes^14–16^, which are also related to NAFLD^17–19^. Smoking would release various hazardous liver-damaging compounds^20^. Continuously reports have associate smoking and the occurrence and development of NAFLD^21–23^. Cigarette smoking worsens nonalcoholic fatty liver disease in obese rats^21^. Furthermore, a study found genetic mutations and NAFLD outbreaks caused by nicotine in electronic cigarettes in mice without apolipoprotein E^22^. An animal investigation revealed that secondhand smoking causes liver steatosis by deregulating genes involved in hepatic lipid metabolism^24^. A large South Korean cohort research based on self-reports, pack-years, and urine cotinine levels found that smoking raises the risk of NAFLD and fibrosis in healthy young and middle-aged adults^25^. Nevertheless, the associations between smoke exposure and hepatic steatosis and fibrosis have still been controversial. For instance, Munsterman et al. reported that smoking increased the degree of NAFLD-related liver fibrosis^26^. Whereas, a limited study indicated that there is no correlation of smoking with the degree of fibrosis in NAFLD patients^27^. The discrepancies in these researches might be attributed to the lack of objective indicators for assessing smoking status, the limited sample amount, and the inability to adequately account for mixed variables.

Although the association of smoking with NAFLD was invested continuously with the prevalence of NAFLD increasing globally, fewer researches focused on the connection between smoking and NAFLD in teenage populations than adults. Therefore, this study sought to evaluate the correlation of serum cotinine levels and the degree of liver fibrosis and hepatic steatosis among teenage participants by analyzed the 2017–2020 data from NHANES. Our findings would indicate the risks of smoke exposure in the adolescent population and offer epidemiologic data for further studies on the relationship of smoke exposure with liver fibrosis and steatosis.

## Methods

### Study population

**Figure 1:**
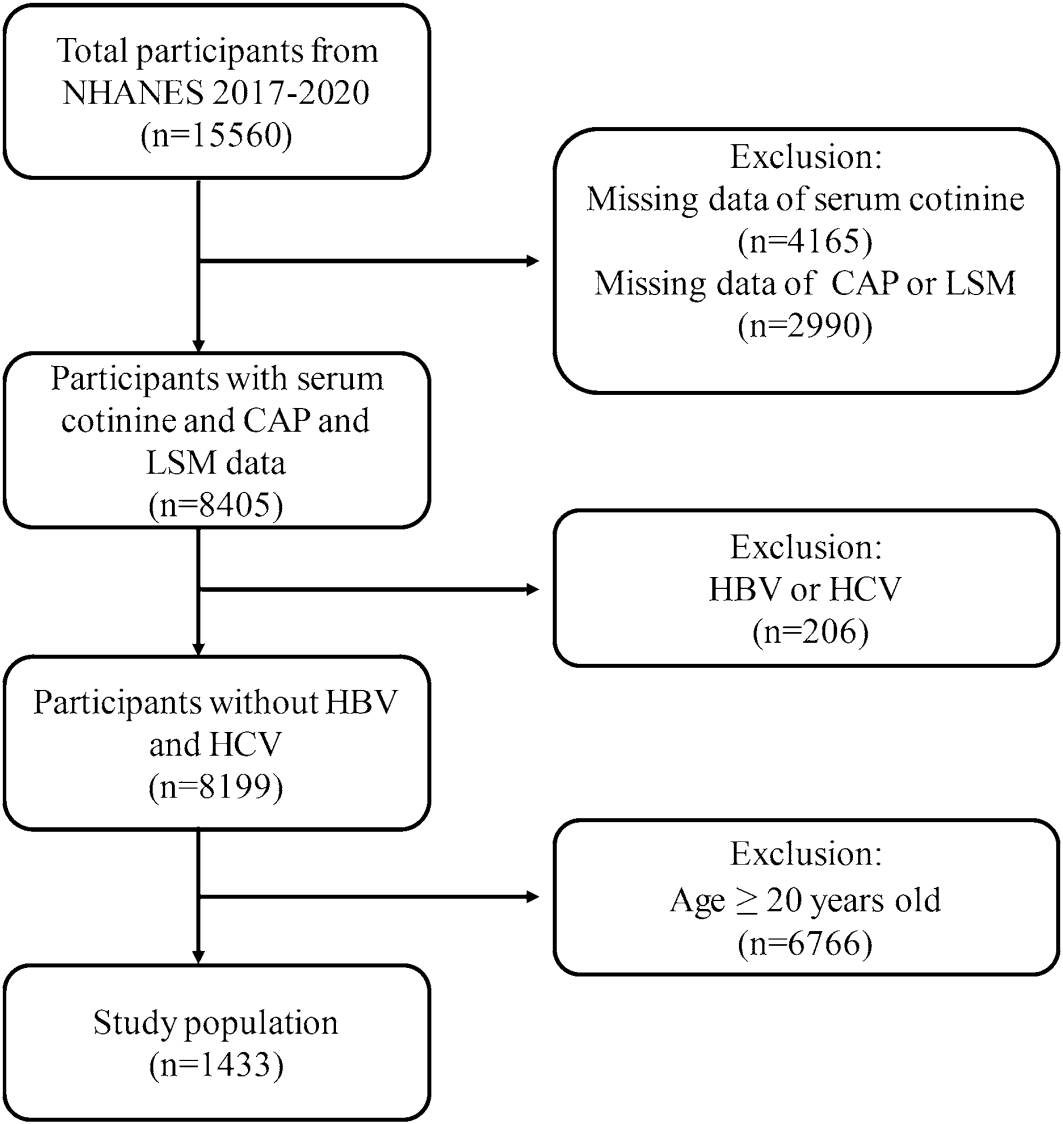
Flowchart of participant selection. *NHANES* National Health and Nutrition Examination Survey, *CAP* controlled attenuation parameter, *LSM* liver stiffness measurement, *HBV* Hepatitis B, *HCV* Hepatitis C.

Data from The National Center for Health Statistics of the Centers for Disease Control and Prevention (https://www.cdc.gov/nchs/nhanes/index.html) were used in the study, which contains data from 2017 to 2020. We removed 4165 patients lacking serum cotinine data, 2990 subjects lacking CAP or LSM data, 206 persons with hepatitis B or C, and ,766 subjects older than 20 from the 15,560 subjects that participated in the study. In the end, 1433 adolescents participated in the study (Fig. 1).

### Study variables

Serum cotinine is a sensitive and specific biomarker to reflect smoking status^28^. Serum cotinine levels can distinguish active and passive smoking^29^. Cotinine, one of the primary nicotine metabolites, is utilized to detect both secondhand smoke exposure and active smoking. Using isotope dilution in combination with atmospheric pressure chemical ionization tandem mass spectrometry and high-performance liquid chromatography, serum cotinine was determined. In brief, methyl-D3-cortinine was added to the serum samples as an internal standard. The samples were alkalized and placed on supported liquid extraction (SLE) plates. The analytes were obtained using isopropanol/dichloromethane, concentrated, and loaded onto a C18 HPLC column. The eluents of these administrations were monitored by APCI-MS/MS. The *m/z* 80 daughter ion of the *m/z* 177 excimer ion was identified as cotinine. In an adolescent group, we used Benowitz's newly proposed threshold of 2.99 ng/ml to distinguish smokers from nonsmokers^30^. Three groups were created based on the cotinine levels: no smoke exposure: ≤ 0.05 ng/ml; passive smokers: 0.05–2.99 ng/ml; and active smokers: > 2.99 ng/ml.

A straightforward, non-invasive ultrasound technique called transient elastography (TE) is used to evaluate the degree of hepatic steatosis and fibrosis in liver disease patients. The FibroScan 502 V2 Touch probe and equipment are used to examine liver ultrasound transient elastography. When a vibrating point comes into contact with skin, it generates a transverse wave via mechanical vibration. This wave travels faster through stiffer tissues and goes through the liver. The liver stiffness measurement (LSM), which is used to assess the degree of liver fibrosis, is calculated from transverse wave velocity and represents the stiffness of the liver. Higher LSM values suggest more extensive liver fibrosis. The controlled attenuation parameter (CAP) evaluates the quantity of fat in the liver and indicates the degree of hepatic steatosis. The research subjects received VCTE. A medium (M) or large (XL) probe was used to collect 30 measurements for each participant. The test was considered reliable if (i) the individual fasted for a minimum of 3 h before the test, (ii) 10 or more complete LSMs were conducted, and (iii) the interquartile range/median of the LSM was below 30%. In this study, LSM and CAP values were used as outcome measures.

### Covariates

The following covariates were present: age, sex, race, the family income to poverty ratio, total cholesterol, direct high-density lipoprotein cholesterol (HDL-C), low-density lipoprotein cholesterol (LDL-C), triglycerides, alanine aminotransferase (ALT), alkaline phosphatase (ALP), aspartate aminotransferase (AST), body mass index (BMI), and serum uric acid. Poverty was assessed using the PIR, the PIR is divided to low-income level (PIR < 1.3) and high-income level (PIR ≥ 1.3). Age, BMI, total cholesterol, HDL-C, ALT, ALP, AST and serum uric acid were analyzed as continuous variables. Other variables were analyzed as categorical variables. All variables have calculations, measurements, and interpretations by visiting the official NHANES website (https://www.cdc.gov/nchs/nhanes/).

### Statistical analysis

Means (standard deviation) were used to determine the continuous variables. Chi-square tests were used to compare the absolute values (percentages) of the categorical variables. Using the log2 transformation, the levels of cotinine were converted to a normal distribution. Additionally, the median was employed to fill in the missing values for < 10% and continuous data were transformed into categorical variables for > 10%. A statistically significant value was defined as *p* < 0.05. The data set's notable volatility was reduced via weighting.

Three separate weighted multivariate regression models were used to examine the relationship between cotinine and hepatic steatosis and liver fibrosis. Model 1 did not alter any of the factors. The model 2 underwent modifications with regards to the variables of race, age, and gender. Age, sex, race, PIR, total cholesterol, HDL-C, LDL-C, triglycerides, ALT, ALP, AST, BMI and serum uric acid was modified in the model 3. The researchers employed various subgroups to conduct statistical analyses. The association between cotinine and LSM in various categories was examined using subgroup analysis and interaction testing. Finally, the association and inflection points between cotinine and liver fibrosis were tested using threshold effect analysis models and smooth curve fitting. The nonlinear relationship of cotinine and LSM was investigated using a weighted generalized additive model and smoothed curve fitting. When nonlinear correlations were found, threshold effects were calculated and each interval was fitted using a two-stage linear regression model, also known as a segmented regression model. All analyses were conducted using Empowerstats (version 4.1) and R (version 4.2).

### Ethics statement

The NHANES survey was approved by the National Center for Health Statistics Institutional Review Board. The study reported in this manuscript was exempt from ethical committee approval because it was based on publicly available data. NHANES has obtained the written informed consent from all participants. All procedures were performed in accordance with relevant guidelines. The studies were conducted in accordance with the local legislation and institutional requirements. The participants provided their written informed consent to participate in this study.

## Results

### Baseline characteristics

The study included 1433 teenagers, whose mean age was 15.44 ± 2.23 years. There were 52.52% men and 47.48% women among these individuals. The average LSM and CAP concentrations were 221.22 ± 53.00 dB/m and 5.01 ± 2.09 kPa, respectively. When subgroup according to cotinine level, 476 (33.22%) individuals showed cotinine levels ranging from 0.05 to 2.99 ng/ml, 775 (54.08%) of the individuals had cotinine levels ≤ 0.05 ng/ml, while 182 (12.70%) had levels > 2.99 ng/ml. Differences in baseline characteristics between cotinine subgroups were significant. Compared with the other subgroups, participants with cotinine levels > 2.99 ng/ml were more likely to be Non-Hispanic White, male, older, and from lower-income backgrounds. Participants in the subgroups of cotinine levels > 2.99 ng/ml also had more significant serum uric acid, lower LDL, lower HDL, lower ALP, and lower BMI (Table 1). Previous research had indicated that among teenagers, older individuals affected by environmental marketing and curiosity had a much greater chance of smoking exposure^31^. Adolescents' exposure to smoke promotes oxidative stress and lipid peroxidation, which leads to a rise in BMI^32^.

**Table 1:** Baseline characteristics of subjects.

Variables	Cotinine category				P value
	Total (n = 1433)	≤ 0.05 (ng/ml)	0.05–2.99 (ng/ml)	> 2.99 (ng/ml)	
Age (years)	15.44 ± 2.23	15.23 ± 2.19	15.31 ± 2.29	16.75 ± 1.74	< 0.001
Sex (%)					< 0.001
Male	52.52	47.45	55.31	71.07	
Female	47.48	52.55	44.69	28.93	
Race (%)					< 0.001
Mexican American	16.61	22.07	8.03	9.94	
Other Hispanic	8.93	9.86	7.76	7.1	
Non-Hispanic White	52.04	48.6	55.44	61.02	
Non-Hispanic Black	11.98	7.84	20.77	11.63	
Other race	10.44	11.63	8	10.31	
PIR					< 0.001
< 1.3	26.26	20.14	36.74	31.7	
≥ 1.3	64.7	70.12	54.17	62.83	
BMI (kg/m^2^)	24.38 ± 6.30	23.73 ± 5.66	25.43 ± 7.03	25.13 ± 6.97	< 0.001
Total Cholesterol (mmol/l)	3.98 ± 0.73	4.00 ± 0.73	3.99 ± 0.74	3.89 ± 0.74	0.206
Triglyceride (mmol/l)					0.294
< 0.5	11.34	11.04	13.27	8.22	
≥ 0.5	34.6	34.83	35.27	31.83	
LDL cholesterol (mmol/l)					0.023
< 2.5	31.19	32.76	29.19	28.21	
≥ 2.5	14.7	13.11	19.21	11.84	
HDL Cholesterol (mmol/l)	1.33 ± 0.30	1.36 ± 0.29	1.30 ± 0.31	1.26 ± 0.33	< 0.001
ALT (U/l)	17.31 ± 19.01	16.73 ± 21.54	17.28 ± 12.46	20.24 ± 18.22	0.088
ALP (IU/l)	146.50 ± 95.21	151.93 ± 98.28	148.10 ± 96.76	115.72 ± 65.62	< 0.001
AST (U/l)	20.12 ± 11.06	20.03 ± 12.08	20.44 ± 9.76	19.81 ± 8.29	0.766
Uric acid (μmol/l)	299.61 ± 75.38	291.07 ± 73.23	304.64 ± 73.98	330.10 ± 80.12	< 0.001
LSM (kPa)	5.01 ± 2.09	4.80 ± 1.53	5.25 ± 2.01	5.47 ± 3.85	< 0.001
CAP (dB/m)	221.22 ± 53.00	217.08 ± 51.46	229.06 ± 54.64	223.09 ± 54.35	< 0.001

*PIR* the family income to poverty ratio, *BMI* body mass index, *HDL-C* high-density lipoprotein cholesterol, *LDL-C* low-density lipoprotein cholesterol, *ALT* alanine aminotransferase, *ALP* alkaline phosphatase, *AST* aspartate aminotransferase, *LSM* Liver Stiffness Measurement, *CAP* Controlled Attenuation Parameter.

### Association between serum cotinine levels and liver fibrosis (LSM)

Multiple regression analyses were utilized to evaluate the relationship between liver fibrosis severity and serum cotinine levels. In the original model, log2-transformed cotinine levels were substantially and positively related to LSM (β = 0.08, 95% CI 0.05–0.11, *p* < 0.001). After adjustment for covariates, the correlation between log2-transformed cotinine levels and LSM was also significant in Model 2 (β = 0.07, 95% CI 0.04–0.10, *p* < 0.001) and Model 3 (β = 0.05, 95% CI 0.03–0.08, *p* < 0.001). When conversion of cotinine transforming a continuous variable into a categorical variable, in the model3, a significant positive correlation between serum cotinine levels > 2.99 ng/ml [β = 0.41 (0.07, 0.76), *p* = 0.018] and 0.05–2.99 ng/ml [β = 0.24 (0.00, 0.49), *p* = 0.048] and LSM significant positive correlation (*p* for trend = 0.006) compared to serum cotinine levels ≤ 0.05 ng/ml (Table 2).

**Table 2:** The association between serum cotinine levels with LSM and CAP.

	Model 1	Model 2	Model 3
LSM β (95% CI)			
Log2-transformed cotinine, ng/ml	0.08 (0.05, 0.11) < 0.001	0.07 (0.04, 0.10) < 0.001	**0.05 (0.03, 0.08) < 0.001**
LBXCOT categories			
≤ 0.05 ng/ml	0	0	0
0.05–2.99 ng/ml	0.45 (0.21, 0.70) < 0.001	0.43 (0.18, 0.67) < 0.001	**0.24 (0.00, 0.49) 0.048**
≥ 2.99 ng/ml	0.67 (0.33, 1.01) < 0.001	0.51 (0.15, 0.86) 0.005	**0.41 (0.07, 0.76) 0.018**
*P* for trend	< 0.001	< 0.001	**0.006**
CAP β (95%CI)			
Log2-transformed cotinine, ng/ml	0.99 (0.29, 1.68) 0.006	0.63 (− 0.09, 1.35) 0.087	− 0.19 (− 0.77, 0.38) 0.512
LBXCOT categories			
≤ 0.05 ng/ml	0	0	0
0.05–2.99 ng/ml	11.98 (5.76, 18.21) < 0.001	11.44 (5.21, 17.67) < 0.001	2.20 (− 2.87, 7.27) 0.395
≥ 2.99 ng/ml	6.01 (− 2.65, 14.67) 0.174	0.82 (− 8.10, 9.75) 0.856	− 3.41 (− 10.56, 3.74) 0.350
*P* for trend	0.006	0.076	0.712

Significant values are in bold.

### Association of serum cotinine levels with hepatic steatosis (CAP)

Log2-transformed cotinine levels were substantially and positively related to in the unadjusted model (β = 0.72, 95% CI 0.29–1.68, *p* = 0.006). After controlling for all covariates, there was no significant connection between log2-transformed serum cotinine levels and CAP (β = − 0.19, 95% CI (− 0.77, 0.38), *p* = 0.512) (Table 2).

### Subgroup analysis

Using stratified weighted multiple regression models stratified by sex, age, and race, we investigated the relationship of cotinine with LSM and CAP in various population situations.

A stronger positive correlation (*p* < 0.05) was found between cotinine and LSM in Mexican American [0.15 (0.08, 0.22)] and Non-Hispanic White [0.04 (0.00, 0.07)] but not in Other Hispanic [0.05 (− 0.06, 0.15)], Non-Hispanic Black [0.01 (− 0.05, 0.07)], and other race [0.09 (− 0.03, 0.21)].Nonetheless, this correlation held for populations in various age and gender grouping (*p* for interaction > 0.05). It was found that the positive association between cotinine and LSM remained significant in the 12–15 years age group [0.06 (0.02, 0.10)] and in the 16–19 years age group [0.06 (0.02, 0.10)]. The positive connection was found between cotinine and LSM in male [0.05 (0.03, 0.08)] but not in female [0.05 (− 0.01, 0.10)]. Furthermore, a noteworthy connection was only identified between cotinine and CAP in the 12–15 years age group [1.07 (0.03, 2.11)] (Table 3).

**Table 3:** Subgroup analysis of the association between serum cotinine levels with LSM and CAP.

Subgroup	LSM β (95%CI)	CAP β (95%CI)
Sex		
Male	0.05 (0.03, 0.08)	0.33 (− 0.35, 1.01)
Female	0.05 (− 0.01, 0.10)	− 0.15 (− 1.20, 0.91)
* P* for interaction	0.97	0.396
Age		
12–15	0.06 (0.02, 0.10)	**1.07 (0.03, 2.11)**
16–19	0.06 (0.02, 0.10)	− 0.25 (− 0.96, 0.45)
* P* for interaction	0.309	**0.004**
Race		
Mexican American	**0.15 (0.08, 0.22)**	− 0.39 (− 2.35, 1.58)
Other Hispanic	0.05 (− 0.06, 0.15)	− 1.35 (− 3.24, 0.53)
Non-Hispanic White	**0.04 (0.00, 0.07)**	0.89 (− 0.03, 1.80)
Non-Hispanic Black	0.01 (− 0.05, 0.07)	0.27 (− 1.18, 1.73)
Other race	0.09 (− 0.03, 0.21)	− 2.19 (− 3.48, − 0.90)
* P* for interaction	**0.007**	0.059

Significant values are in bold.

### Nonlinear relationship between serum cotinine levels with liver fibrosis

**Figure 2:**
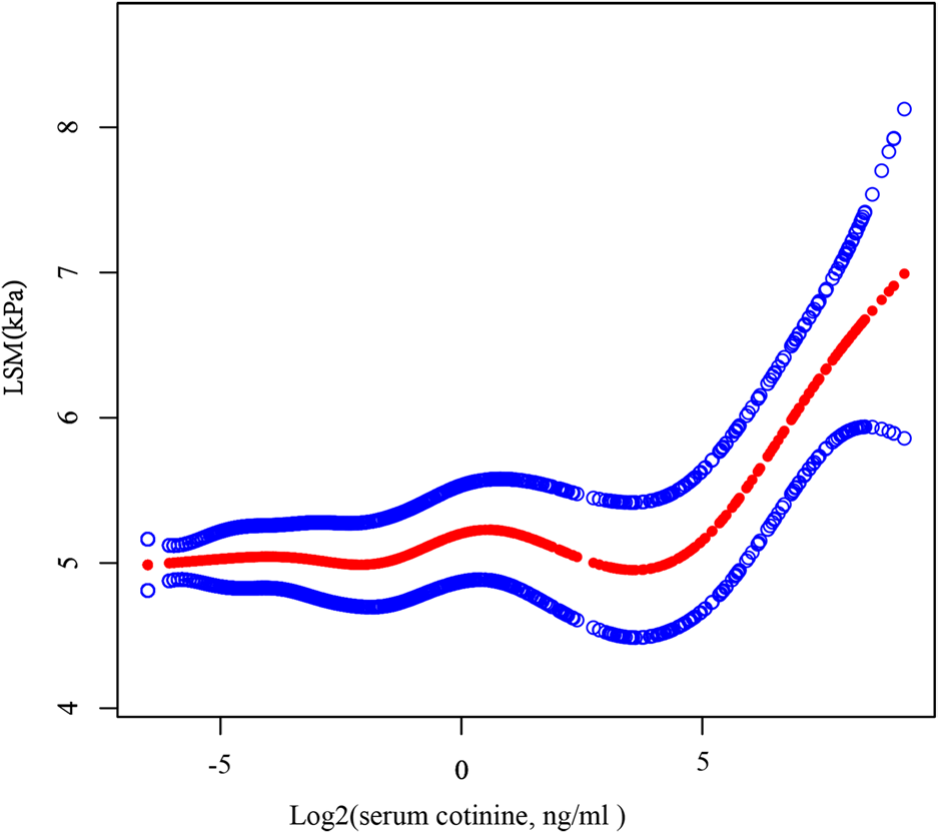
The association between serum cotinine and LSM. The solid red line represents the smooth curve fit between variables. Blue bands represent the 95% confidence interval from the fit.

After considering all variables, it was found that a nonlinear correlation exists between LSM and log2-transformed cotinine levels used the smooth curve fitting and the generalized additive model (Fig. 2). Cotinine levels that were log2-transformed and LSM displayed a U-shaped relationship (inflection point: 4.53). To be more precise, LSM showed a positive correlation with cotinine > 4.53 [β = 0.29, 95% CI (0.10, 0.47), *p* = 0.002] and a negative correlation with cotinine < 4.53 [β = 0.02, 95% CI (− 0.02, 0.06), *p* = 0.268] (Table 4).

**Table 4:** Threshold effect analysis of serum cotinine levels on LSM using two-piecewise linear regression model.

Log2-transformed cotinine	LSM adjusted β (95% CI) *p* value
Fitting by the standard linear model	0.05 (0.03, 0.08) < 0.001
Fitting by the two-piecewise linear model	
Inflection point	4.53
< K segment effect	0.02 (− 0.02, 0.06) 0.268
> K segment effect	0.29 (0.10, 0.47) 0.002
Log likelihood ratio	0.012

## Discussion

In this research, we observed a statistically significant positive relationship between smoking and an increased risk of liver fibrosis, but not hepatic steatosis. After the adjustment for multiple covariates, log2-transformed serum cotinine levels revealed a dose–response favorable association with liver fibrosis (p < 0.001), but no significant link with hepatic steatosis was discovered (p = 0.512). There was an obvious tendency toward higher serum cotinine levels with higher liver fibrosis. We discovered an approximate U-shaped relationship with an inflection point of 4.53 between log2-transformed cotinine levels and LSM. Subgroup studies revealed that Mexican American and Non-Hispanic White participants had a stronger positive connection between cotinine and LSM. Moreover, in the population between the ages of 12 and 15, a strong positive connection between cotinine and CAP was noted. We found, quite interestingly, men are more affected than women. The positive connection was found between cotinine and LSM in male but not in female. Similarly, a positive association between current smoking and NAFLD risk among men but not among women was reported^25^. This predisposition to develop NAFLD in males rather than females may be attributed to estrogen, which is protecting throughout the reproductive time^33^.

Growing evidences show that smoking is closely related to the growth of many diseases, including NAFLD. Existing research has indicated a strong association between smoking and the onset of liver fibrosis and hepatic steatosis^20,34–36^. However, our research indicated that youth smoking exposure is associated with liver fibrosis but not hepatic steatosis. Furthermore, a study on passive smoking revealed that, based on data from parents who also smoke, adults who are exposed to secondhand smoke as youngsters are more likely to develop fatty livers^37^. Nevertheless, several studies have demonstrated that there are no distinctions between current smokers and nonsmokers in the prevalence or severity of NAFLD^27,38–40^. The majority of research has employed self-reported questionnaires to ascertain participants' smoking status, various factors would lead to a lack of objectivity in the questionnaire results, which may account for the discrepant conclusions. Furthermore, a sizable US population-based investigation revealed no correlation of NAFLD with either self-reported smoking status or serum cotinine levels^41^. This conclusion may be constrained by the sample size. To assure the accuracy of the results, we use vast samples from NHANES. Due to the lack of clinical data and the contradictory findings, the consequence of smoking exposure on NAFLD in humans has been a contentious topic. this study used a serum cotinine as an indicator of tobacco exposure in the environment to reflect the individual's level of smoking objectively in epidemiological research^42^. In this study, we accurately differentiate active and passive smoking populations based on Benowitz et al. reported optimal cotinine thresholds^30^.To increase the accuracy of the results, the relationships of serum cotinine levels and LSM and CAP were not only evaluated in the adolescent population while accounting for the effects of multiple confounders but they were also categorized to evaluate the associations in the populations of smokers who were passive and active. Not just active smoking but also passive smoking has been linked to NAFLD in several studies^24,43^. Our research showed that adolescents who smoked actively or were exposed to secondhand smoke were more likely to develop liver fibrosis and these associations held regardless of the participants' age or gender.

The mechanism underlying the association of serum cotinine with liver fibrosis and steatosis is still uncertain. In addition, smoking associates with fibrosis has also been shown in other organs^44,45^. Rockey et al. reported that cotinine, one of nicotine's main byproducts, would activate fibroblast and strengthen pro-fibrotic processes^46^. Moreover, nicotine could activate nicotinic acetylcholine receptors (nAChR) to stimulate hepatic injury and subsequent fibrogenesis^47^. Other numerous theories gave credit to the rise of transforming growth factors-β (TGF-β), proinflammatory cytokines, and inflammatory cells brought on by smoking^48,49^. As a result, non-proliferative and inactive hepatic stellate cells (HSCs) were stimulated and differentiated into myofibroblasts, resulting in increased fibrillar ECM protein production, collagen I and III depositions in the Disse region, and fast fibrosis development. Muriel et al. found that smoking will activate NADPH oxidase and increase the production of reactive oxygen species (ROS), which worsens hepatocyte damage, lipid accumulation, and oxidative stress^50^. Other study suggests that smoking induces intestinal dysbiosis and bacterial translocation, activating the Toll-like receptor 4 on HSCs, leading to cell activation and fibrosis^51^.

This study has several limitations. Firstly, we could not determine a causal correlation of serum cotinine with CAP and LSM because of the nature of cross-sectional studies. Secondly, the concentrations of cotinine were determined by NHANES using a single serum sample, which does not represent long-term smoke exposure. Furthermore, even after accounting for several confounders, we could not completely rule out the impact of additional confounding variables, which impacted the precision of the findings. On the other hand, our research quantifies smoke exposure as indicated by serum cotinine for the first time and is based on a sizable cross-sectional survey. Second, we measured liver stiffness using transient elastography. Transient elastography is a straightforward, noninvasive, and precise method regarded as a noninvasive standard tool for evaluating liver fibrosis. CAP score is another feature of transient elastography used to measure ultrasonic attenuation related to hepatic steatosis.

## Conclusion

Our research shows that increased serum cotinine in an American adolescent population is correlated to hepatic fibrosis but not hepatic steatosis. Our study emphasizes the importance of active and passive smoking in the advancement of hepatic steatosis and cirrhosis and the need to limit youth exposure to smoke to prevent NADPH. More extensive prospective studies are required to validate our results.

## Data Availability

The datasets supporting the conclusions of this article are available in the 2017–2020 continuous National Health and Nutrition Examination Survey: https://www.cdc.gov/nchs/nhanes/index.html.

## References

[1] Mann JP, Valenti L, Scorletti E, Byrne CD, Nobili V (2018). Nonalcoholic fatty liver disease in children. Semin. Liver Dis..

[2] Rich NE, Noureddin M, Kanwal F, Singal AG (2021). Racial and ethnic disparities in non-alcoholic fatty liver disease in the USA. Lancet. Gastroenterol. Hepatol..

[3] Anderson EL (2015). The prevalence of non-alcoholic fatty liver disease in children and adolescents: A systematic review and meta-analysis. PLoS One.

[4] Kim M, Kim J (2022). Cardiometabolic risk factors and metabolic syndrome based on severity of obesity in Korean children and adolescents: Data from the Korea National Health and Nutrition Examination Survey 2007–2018. Ann. Pediatr. Endocrinol. Metab..

[5] Díaz LA (2022). The establishment of public health policies and the burden of non-alcoholic fatty liver disease in the Americas. Lancet. Gastroenterol. Hepatol..

[6] Ginès P (2021). Liver cirrhosis. Lancet.

[7] Eslam M (2020). A new definition for metabolic dysfunction-associated fatty liver disease: An international expert consensus statement. J. Hepatol..

[8] Imajo K (2016). Magnetic resonance imaging more accurately classifies steatosis and fibrosis in patients with nonalcoholic fatty liver disease than transient elastography. Gastroenterology.

[9] Castera L, Friedrich-Rust M, Loomba R (2019). Noninvasive assessment of liver disease in patients with nonalcoholic fatty liver disease. Gastroenterology.

[10] Ciardullo S, Monti T, Perseghin G (2021). High prevalence of advanced liver fibrosis assessed by transient elastography among U.S. adults with type 2 diabetes. Diabetes Care.

[11] Siddiqui MS (2019). Vibration-controlled transient elastography to assess fibrosis and steatosis in patients with nonalcoholic fatty liver disease. Clin. Gastroenterol. Hepatol..

[12] Shaunak M (2021). Non-alcoholic fatty liver disease and childhood obesity. Arch. Dis. Child..

[13] Hou W (2023). Associations between smoke exposure and osteoporosis or osteopenia in a US NHANES population of elderly individuals. Front. Endocrinol..

[14] Jatoi NA, Jerrard-Dunne P, Feely J, Mahmud A (2007). Impact of smoking and smoking cessation on arterial stiffness and aortic wave reflection in hypertension. Hypertension (Dallas, Tex.: 1979).

[15] Botteri E (2008). Smoking and colorectal cancer: A meta-analysis. JAMA.

[16] Willi C, Bodenmann P, Ghali WA, Faris PD, Cornuz J (2007). Active smoking and the risk of type 2 diabetes: A systematic review and meta-analysis. JAMA.

[17] Kasper P (2021). NAFLD and cardiovascular diseases: A clinical review. Clin. Res. Cardiol..

[18] Tanase DM (2020). The intricate relationship between type 2 diabetes mellitus (T2DM), insulin resistance (IR), and nonalcoholic fatty liver disease (NAFLD). J. Diabetes Res..

[19] Marengo A, Rosso C, Bugianesi E (2016). Liver cancer: Connections with obesity, fatty liver, and cirrhosis. Annu. Rev. Med..

[20] Kim NH (2018). Association between cotinine-verified smoking status and risk of nonalcoholic fatty liver disease. Liver Int..

[21] Azzalini L (2010). Cigarette smoking exacerbates nonalcoholic fatty liver disease in obese rats. Hepatology (Baltimore, Md.).

[22] Hasan KM (2019). E-cigarettes and western diet: Important metabolic risk factors for hepatic diseases. Hepatology (Baltimore, Md.).

[23] Yuan H, Shyy JY, Martins-Green M (2009). Second-hand smoke stimulates lipid accumulation in the liver by modulating AMPK and SREBP-1. J. Hepatol..

[24] Tommasi S, Yoon JI, Besaratinia A (2020). Secondhand smoke induces liver steatosis through deregulation of genes involved in hepatic lipid metabolism. Int. J. Mol. Sci..

[25] Jung H-S (2019). Smoking and the risk of non-alcoholic fatty liver disease: A cohort study. Off. J. Am. Coll. Gastroenterol. ACG.

[26] Munsterman ID (2017). Smoking is associated with severity of liver fibrosis but not with histological severity in nonalcoholic fatty liver disease. Results from a cross-sectional study. Scand. J. Gastroenterol..

[27] Yilmaz Y, Yonal O, Kurt R, Avsar E (2010). Cigarette smoking is not associated with specific histological features or severity of nonalcoholic fatty liver disease. Hepatology.

[28] Benowitz NL (2020). Biochemical verification of tobacco use and abstinence: 2019 update. Nicotine Tob. Res..

[29] Kim S (2016). Overview of cotinine cutoff values for smoking status classification. Int. J. Environ. Res. Public Health..

[30] Benowitz NL, Bernert JT, Caraballo RS, Holiday DB, Wang J (2008). Optimal serum cotinine levels for distinguishing cigarette smokers and nonsmokers within different racial/ethnic groups in the United States between 1999 and 2004. Am. J. Epidemiol..

[31] Wang TW (2019). Tobacco product use and associated factors among middle and high school students—United States, 2019. MMWR Surveill. Summ..

[32] Kaur J, Upendra S, Barde S (2024). Inhaling hazards, exhaling insights: A systematic review unveiling the silent health impacts of secondhand smoke pollution on children and adolescents. Int. J. Environ. Health Res..

[33] Mumtaz, H., Hameed, M., Sangah, A. B., Zubair, A. & Hasan, M. Association between smoking and non-alcoholic fatty liver disease in Southeast Asia. *Front. Public Health.* **10** (2022). 10.3389/fpubh.2022.1008878 PMC9793992 36582387

[34] Jung HS (2019). Smoking and the risk of non-alcoholic fatty liver disease: A cohort study. Am. J. Gastroenterol..

[35] Ma N (2023). Environmental exposures are important risk factors for advanced liver fibrosis in African American adults. JHEP Rep..

[36] Jang TY (2023). Air pollution associate with advanced hepatic fibrosis among patients with chronic liver disease. Kaohsiung J. Med. Sci..

[37] Wu F (2021). Childhood and adulthood passive smoking and nonalcoholic fatty liver in midlife: A 31-year cohort study. Am. J. Gastroenterol..

[38] Chavez-Tapia NC, Lizardi-Cervera J, Perez-Bautista O, Ramos-Ostos MH, Uribe M (2006). Smoking is not associated with nonalcoholic fatty liver disease. World J. Gastroenterol..

[39] Singh A, Lopez R, Lawitz E, Poordad F, Alkhouri N (2017). Association of smoking with advanced fibrosis in type 2 diabetics with suspected non-alcoholic fatty liver disease: 997. Am. J. Gastroenterol..

[40] Haenle MM (2006). Overweight, physical activity, tobacco and alcohol consumption in a cross-sectional random sample of German adults. BMC Public Health.

[41] Shen H, Peng JL, Tayarachakul S, Liangpunsakul S (2017). Association between serum cotinine level and prevalence of non-alcoholic fatty liver disease: A cross-sectional study from the Third National Health and Nutrition Examination Survey. J. Investig. Med..

[42] Seccareccia F (2003). Serum cotinine as a marker of environmental tobacco smoke exposure in epidemiological studies: The experience of the MATISS project. Eur. J. Epidemiol..

[43] Lin C (2014). Secondhand tobacco exposure is associated with nonalcoholic fatty liver disease in children. Environ. Res..

[44] Jensen K (2013). Chronic nicotine exposure stimulates biliary growth and fibrosis in normal rats. Dig. Liver Dis..

[45] van Geenen EJ (2011). Smoking is related to pancreatic fibrosis in humans. Am. J. Gastroenterol..

[46] Rockey DC, Bell PD, Hill JA (2015). Fibrosis—A common pathway to organ injury and failure. N. Engl. J. Med..

[47] Jensen K (2012). General mechanisms of nicotine-induced fibrogenesis. FASEB J..

[48] Soeda J (2012). Nicotine induces fibrogenic changes in human liver via nicotinic acetylcholine receptors expressed on hepatic stellate cells. Biochem. Biophys. Res. Commun..

[49] Yang SR (2006). Cigarette smoke induces proinflammatory cytokine release by activation of NF-kappaB and posttranslational modifications of histone deacetylase in macrophages. Am. J. Physiol. Lung Cell Mol. Physiol..

[50] Muriel P (2009). Role of free radicals in liver diseases. Hepatol. Int..

[51] Zuo L (2014). Cigarette smoking is associated with intestinal barrier dysfunction in the small intestine but not in the large intestine of mice. J. Crohns Colitis..

